# 3D-Printed PLA-Bioglass Scaffolds with Controllable Calcium Release and MSC Adhesion for Bone Tissue Engineering

**DOI:** 10.3390/polym14122389

**Published:** 2022-06-13

**Authors:** Eva Schätzlein, Christoph Kicker, Nicolas Söhling, Ulrike Ritz, Jonas Neijhoft, Dirk Henrich, Johannes Frank, Ingo Marzi, Andreas Blaeser

**Affiliations:** 1 Institute for BioMedical Printing Technology, Technical University of Darmstadt, 64289 Darmstadt, Germany; schaetzlein@idd.tu-darmstadt.de; 2 Technical University of Darmstadt, 64289 Darmstadt, Germany; christoph.kicker@stud.tu-darmstadt.de; 3 Department of Trauma, Hand and Reconstructive Surgery, Goethe University Frankfurt am Main, 60323 Frankfurt am Main, Germany; nicolas.soehling@kgu.de (N.S.); jonas.neijhoft@kgu.de (J.N.); d.henrich@trauma.uni-frankfurt.de (D.H.); johannes.frank@kgu.de (J.F.); marzi@trauma.uni-frankfurt.de (I.M.); 4 BiomaTiCS Group, Department of Orthopaedics and Traumatology, University Medical Center, Johannes Gutenberg University Mainz, 55122 Mainz, Germany; ritz@uni-mainz.de; 5 Centre for Synthetic Biology, Technical University of Darmstadt, 64289 Darmstadt, Germany

**Keywords:** bone tissue engineering, cell seeding, biofabrication, fused filament fabrication, 3D printing, bioactive glass, polymer ceramic composites, PLA bioglass

## Abstract

Large bone defects are commonly treated by replacement with auto- and allografts, which have substantial drawbacks including limited supply, donor site morbidity, and possible tissue rejection. This study aimed to improve bone defect treatment using a custom-made filament for tissue engineering scaffolds. The filament consists of biodegradable polylactide acid (PLA) and a varying amount (up to 20%) of osteoconductive S53P4 bioglass. By employing an innovative, additive manufacturing technique, scaffolds with optimized physico-mechanical and biological properties were produced. The scaffolds feature adjustable macro- and microporosity (200–2000 µm) with adaptable mechanical properties (83–135 MPa). Additionally, controllable calcium release kinetics (0–0.25 nMol/µL after 24 h), tunable mesenchymal stem cell (MSC) adhesion potential (after 24 h by a factor of 14), and proliferation (after 168 h by a factor of 18) were attained. Microgrooves resulting from the 3D-printing process on the surface act as a nucleus for cell aggregation, thus being a potential cell niche for spheroid formation or possible cell guidance. The scaffold design with its adjustable biomechanics and the bioglass with its antimicrobial properties are of particular importance for the preclinical translation of the results. This study comprehensibly demonstrates the potential of a 3D-printed bioglass composite scaffold for the treatment of critical-sized bone defects.

## 1. Introduction

Native bone usually possesses good healing properties. However, for large bone defects above the critical size of about 2.5 cm, the endogenous regenerative capacity of bone tissue is not sufficient for self-repair [1,2].

Commonly used methods for treatment of large bone defects are replacement with autografts and allografts. These are associated with considerable drawbacks: limited supply, donor site morbidity, possibility of tissue rejection, and transmission of disease [1,2,3,4]. The combination of bioactive substances and biodegradable polymers processed by additive manufacturing presents itself as an improved method for the treatment of bone defects [5,6,7,8,9].

Biopolymers routinely used in 3D printing, such as polylactide acid (PLA), polycaprolactone (PCL) and polylactic-co-glycolic acid (PLGA) have shown to be well suited for the treatment of bone defects due to their processing ease and generation of non-toxic, resorbable degradation products [5,8,10,11]. PLA in particular offers excellent processability for 3D printing, but can lead to inflammation due to acidic degradation. Without additives or further treatment, PLA exhibits low bioactivity, a hydrophobic surface, and a slow rate of degradation, which can hinder the healing process [4,10].

Possible additives for these biopolymers are bioactive ceramics and glasses, e.g., hydroxy carbonate apatite (HCA), hydroxy apatite, tricalcium phosphate, and bioglass, as they are an alternative for the treatment of bone defects. These additives offer bone-like chemistry, good cell attachment, and porous surfaces [4]. Bioglass exhibits faster formation of HCA, by adsorption of Ca^2+^, PO_4_, and CO_3_^2+^ to the Silica gel surface [11,12], than ceramics, and thus promotes osteoconductivity [13,14,15].

Furthermore, bioglass can suppress the growth of many, even multi-resistant, bacterial strains [13,16]. SP53P4 Bioglass in particular, hinders the growth of anaerobe bacteria exceptionally well [14]. However, the fabrication of structures from bioglass is complex and requires solvents or high-temperature sintering [11]. Therefore, it is very difficult to create the highly complex hierarchical structures needed for bone tissue engineering [15]. Furthermore, its low mechanical properties, such as brittleness and low fracture toughness [17,18], limit the applications of bioglass, and due to its high thermal expansion coefficient, coatings of bioglass on various metallic implants are not ideal [8,19,20].

The combination of bioactive substances with biodegradable polymers shows promising results for the treatment of large bone defects [6,7,8,19,21,22]. It was shown in an animal model that S53P4 bioglass scaffolds can induce osteosimulative and osseointegrative new bone formation in critical-sized defects [23]. However, manufacturing approaches differ considerably. For example, some groups use paste-like combinations of chloroform solvent, PCL, and borate bioglass [7] or mixtures of chloroform solvent, PLA and calcium-phosphate glass using polyethylenglycol (PEG) as a plasticizer [6]. Following evaporation of the solvent, fine structures with high bioglass content of up to 50% were achieved [7].

Another approach is to mix the ingredients and melt them together [8,24,25]. The composite is either used as pellets [20] or as a filament [8,21]. The latter was utilized for fused filament fabrication (FFF) printing for example with a PLA/bioglass filament to produce simple lattice geometries with lower bioglass contents of up to 10% [8,21].

FFF printing offers several advantages over paste-based approaches since it can be processed without solvents, is storable, and allows for easy quality control [8]. In addition, the printing technique allows the material to be patterned with a pore size gradient [24]. For example, smaller pores in the range of 10 µm to 200 µm promote protein and cell adhesion as well as osteointegration, leading to improved new bone formation [17,24], while larger pores greater than 300 µm support cell infiltration from surrounding tissue, which promotes vascularization [17,24].

The present work aimed to combine S53P4 bioglass with biodegradable PLA to fabricate delicate structures with sub-nozzle diameter resolution. In contrast to previous work, the PLA was supplemented with up to 20% bioglass without limiting its printability using fused filament fabrication. 3D printed geometries were produced that were specifically designed to support bone and vessel formation with multiple pore sizes. In addition, designs were chosen that resemble the biological geometry of bone with a thick cortical bone lining and a highly porous filling resembling cancellous bone. The mechanical properties of the composite and printed structures were analyzed. The composite’s bio-chemical properties were compared to native PLA with respect to its calcium release and the proliferation potential of mesenchymal stem cells (MSC) were investigated, both known to be important indicators for improved osteogenic regeneration [26].

## 2. Materials and Methods

### 2.1. Filament Fabrication

Composite filaments of PLA and bioglass were fabricated using PLA granules (PLA-filament Kristall Natur, 3dk.berlin, Berlin, Germany) with a grain size of 2–5 mm and bioglass Type S53P4 (bioglass composition: 53% SiO_2_, 23% Na_2_O, 20% CaO, 4% P_2_O_5_, BonAlive Biomaterials Ltd., Turku, Finland) with a grain size of 25–42 µm. To generate the PLA granules from the starting material, PLA filament was dried at 40 °C for at least 12 h before being shredded (M 50/80, Hellweg Maschinenbau, Roetgen, Germany). Particles that were too large were sorted out with a 3D printed screen with a pore size of 2.9 × 2.25 mm. PLA and bioglass grains were mixed manually to obtain starting materials with bioglass contents of 0%, 5%, 10%, and 20% (*w*/*w*). A desktop filament extruder (NEXT 1.0 Advanced, 3devo B.B., Utrecht, Netherlands) was used for filament extrusion. To prevent material agglomeration, the PLA-bioglass mixture was filled into the hopper in small portions. Pure PLA granules were used to create a stable extruding process when feeding and removing the PLA-bioglass mixture. The screw speed was set to 4 U/min and the fan speed to 65%. To achieve the desired filament diameter of 1.75 mm, the speed of the conveying mechanism was set to automatic. The manufactured composite filaments were classified as BG-0, BG-5, BG-10, and BG-20 for starting materials with 0%, 5%, 10%, and 20% (*w*/*w*) bioglass.

### 2.2. Sample Fabrication

Cylindrical scaffolds of each PLA-bioglass composite were printed using FFF on a 3D printer (i3 MK3S, Prusa Research, Prague, Czech Republic) with a nozzle diameter of 0.4 mm. Filaments were dried at 40 °C for at least 12 h. The scaffolds were designed with the computer-aided design software NX 12 (Siemens NX 12, Siemens AG, Berlin and Munich, Germany) and preprocessed with Cura Ultimaker v.4.6. (Ultimaker, Utrecht, The Netherlands). Flat cylinders with a 5 mm diameter and 0.3 mm height, and porous scaffolds with a 6 mm height, 5 mm diameter, and 1.5 mm wall thickness, were printed. Macroporosity was achieved by designing holes of 700 µm diameter in the wall, and interconnected porosity was produced by the fabrication process that used a line distance of approximately 150 µm between the extruded filaments. Fine filament strands with a diameter below the nozzle diameter were manufactured using a printing technique described previously [27], with a printing speed of 10 mm/s, material flow of 70%, printing temperature of 225 °C, infill line distance of 0.4 mm, and infill line width of 0.3 mm.

### 2.3. Filament Diameter

The filament diameter was measured continuously by the internal measurement of the extruder and with a digital caliper (WZ0031, LogiLink, Schalksmühle, Germany) at 10 points with 10 cm distance in between.

### 2.4. Imaging

For pore and printing quality evaluation, the 3D printed scaffolds were immersed in liquid nitrogen and cut in half. The top layer and cutting surfaces were investigated using a light microscope (DM4000M, Leica Microsystems, Wetzlar, Germany).

The particle distribution of bioglass in the composite filament and the 3D-printed scaffolds was assessed using scanning electron microscopy (SEM) (Zeiss EVO 10—SmartSEM touch, Carl-Zeiss AG, Jena, Germany). Images were acquired in variable pressure mode without sputtering using the backscatter detector. Adjustable parameters were selected as follows: voltage of 20 kV, current of 500 pA, pressure of 70 Pa, and magnification in a range of about 100.

### 2.5. Image Analysis

Microscopic images were analyzed using the free software ImageJ (Fiji v1.53f51, Wayne Rasband and contributors, National Institute of Health, Bethesda, MD, USA). Bioglass particles were detected using a color threshold and the analyze particles function. The result was used to calculate the particle area. The distance from a particle’s center to its nearest neighbor was calculated using the Nearest Neighbor Distances Calculation plugin (https://icme.hpc.msstate.edu/mediawiki/index.php/Nearest_Neighbor_Distances_Calculation_with_ImageJ.html, accessed on 1 October 2021).

### 2.6. Assessment of Mechanical Properties

The mechanical properties of filaments and scaffolds were determined using a static material testing machine (Z050, ZwickRoell, Ulm, Germany). Environmental conditions were kept stable at 23 °C and 50% humidity according to DIN EN 20187. The mechanical tensile properties of PLA-bioglass composite filaments were determined via uniaxial tensile testing. Specimens of 30 mm length were mounted using a 1 kN load cell, an initial load of 1 N, and constant deformation speed of 10 mm/s according to DIN 53455. To assess mechanical deformation behavior of the 3D printed porous scaffolds, compression strength tests were performed with a 1 kN load cell and a constant deformation speed of 5 mm/s with initial load of 0.2 N.

### 2.7. Calcium Release

Specimens of BG-0, BG-5, BG-10, or BG-20 were each placed in individual wells of a 96-well plate and incubated with 200 µL of phosphate-buffered saline (PBS) without calcium and magnesium for 1 h, 2 h, 4 h, 8 h, 24 h, 72 h, or 168 h. The supernatants were removed at the end of the incubation periods. The calcium content in the supernatants was determined by a colorimetric assay according to the manufacturer’s instructions (Sigma-Aldrich, Taufkirchen, Germany) using a plate photometer (Tecan, Mannedorf, Switzerland) at a wavelength of 575 nm. The conversion of absorbance values to calcium concentrations in nmol/µL was performed using a calcium standard curve according to the manufacturer’s instructions.

### 2.8. Ethics

An MSC pool was obtained from residual bone marrow samples from 5 healthy donors. The use for research purposes is covered by an ethics vote (329/10 of the department of medicine of the Goethe University). All donors signed informed consent.

### 2.9. Establishment and Characterization of the MSC Pool

Pooled MSCs were used in this study to minimize the influence of differences between individuals. Mononuclear cells were isolated by ficoll density gradient centrifugation from EDTA-anti-coagulated bone marrow samples (approximately 1–2 mL volume) as previously described [25]. Mononuclear cells were seeded at a density of 1 × 10^5^ cells/cm^2^ using MesenCult + supplements (hereinafter referred to as ‘complete medium’, Stemcell Technologies, Cologne, Germany) and cultured in 75 cm^2^ culture flasks for two additional passages after reaching confluence. Cells were then harvested, and 1 × 10^6^ MSCs per 1 mL of freezing medium (consisting of 90% fetal calf serum and 10% dimethyl sulfoxide) was stored in liquid nitrogen until the target number of donors was reached. To create the pool, one vial of MSCs from each donor was thawed and cultured in complete medium over another passage. Cells were then enzymatically detached and counted. 1 × 10^6^ cells from each donor were pooled, centrifuged, resuspended in freezing medium, and stored in liquid nitrogen until later use. Pooled MSCs were cultured after thawing for one further passage in complete medium and used for either characterization or experiments.

### 2.10. Adhesion of MSCs

The composite samples were placed into individual wells on a 12-well plate. 1 × 10^4^ MSCs per volume of 15 µL were carefully loaded into the test bodies. A 30 min incubation period at 37 °C and 100% humidity allowed the cells to adhere to the material. Each well was carefully filled with 500 µL of medium, and the cells were then incubated at 37 °C for 24 h, 72 h or 168 h. To identify the cells directly on top of the specimens, the cells were stained with Calcein-AM (BD-Biosciences, Heidelberg, Germany). This required removing the former medium and carefully adding 1 mL of fresh prewarmed medium containing 20 µM Calcein-AM to each well, followed by incubating at 37 °C for 40 min. After washing three times with PBS, the cells were counterstained with DAPI (1 μg/mL in PBS, 10 min, Sigma-Aldrich, Taufkirchen, Germany). After three more washes with PBS, the specimens were analyzed using a fluorescence microscope (Axioobserver Z1, Zeiss, Gottingen, Germany). Using three fields of view at 100× magnification, the cells were counted and a mean value was calculated for each sample. The experiment was performed three times.

### 2.11. Statistical Analysis

Data in this work are presented as means ± standard deviations of the mean (SD) unless noted otherwise. For comparing two groups, a two tailed Student’s *t*-test assuming unequal variances was applied using Microsoft Excel (Microsoft Corporation, Redmond, WA, USA). A Kruskal–Wallis Test followed by a Bonferroni–Holm adjusted Conover Iman-posthoc test was performed to analyze cell adherence on the test specimen. The software used was Bias 206 11.12 (Epsilon-Verlag, Darmstadt, Germany). Results were considered significant for values of *p* < 0.05.

## 3. Results

This study investigated the use of a custom-made PLA bioglass filament for 3D-printing of highly porous scaffolds for bone tissue engineering.

### 3.1. Filament Extrusion

**Figure 1:**
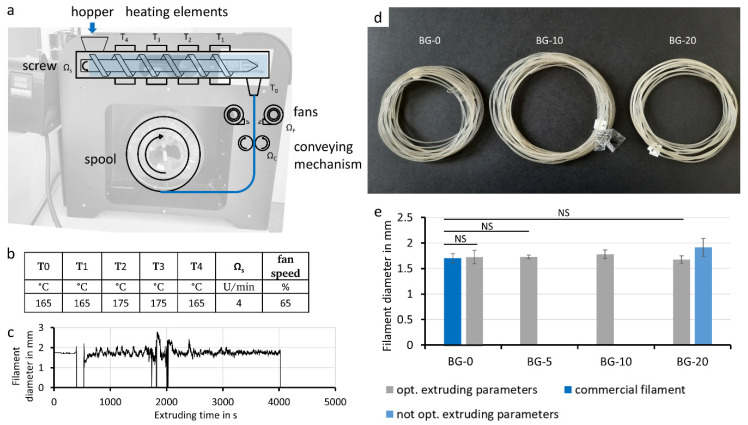
PLA-bioglass composite filament extrusion. Filament extruder with parameters important for filament extrusion (**a**). Optimized parameters used for filament extrusion: temperature zones T0–T4, screw rotation Ωs, and fan speed (**b**). The conveying mechanism was set to automatic and was responsible for the resulting filament diameter, which is shown for the first 4000 s of one batch (**c**). The produced composite filaments BG-0, BG-10, and BG-20 with bioglass contents of 0%, 10%, and 20% in the starting material (**d**). An overview of filament diameters for a commercial filament, filaments manufactured with optimized extruding parameters, and a filament manufactured with non-optimized extruding parameters are shown (**e**). (Mean ± SD, *n* = 10, NS indicates not significant differences of *p* > 0.05).

As a first step, the PLA bioglass filament was extruded (Figure 1a). This process involves mixing the PLA granules and the bioglass particles and loading them into the hopper. The mixture passes through several temperature zones and is extruded. The filament diameter is primarily controlled by the speed of the subsequent conveying step. The material composition (bioglass content, particle size, humidity) and extruder settings (temperature, load volume, extrusion speed, fan speed, extruding time) (Figure 1b) have been suspected to influence the filaments’ diameter, morphology and mechanical properties. As a parameter of quality, the diameter analysis is shown (Figure 1c). The resulting diameter, tracked with the built-in diameter measurement of the extruder in relation to the extruding time, showed several variations from the set diameter of 1.75 mm (Figure 1c). Drops to zero are usually caused by the filament detaching from the conveying mechanism, where it cannot be measured. The beginning showed high variations, demonstrating the need for time to achieve a stable extruding process.

With the presented optimized extruding parameters BG-0, BG-5, BG-10, and BG-20, filament with bioglass content of the starting material of 0%, 5%, 10%, and 20% (*w*/*w*), respectively, were achieved (Figure 1d). The filament transparency decreased with increasing bioglass content.

Filaments extruded with optimized extruding parameters have a similar diameter variation around the target value of 1.75 mm as the commercial filament irrespective of the bioglass content (Figure 1e). The filament extruded with not-optimized parameters and the BG-10 filament exhibited a higher deviation from the target value and differed significantly from the commercial filament.

### 3.2. Composite Filament Characterization

**Figure 2:**
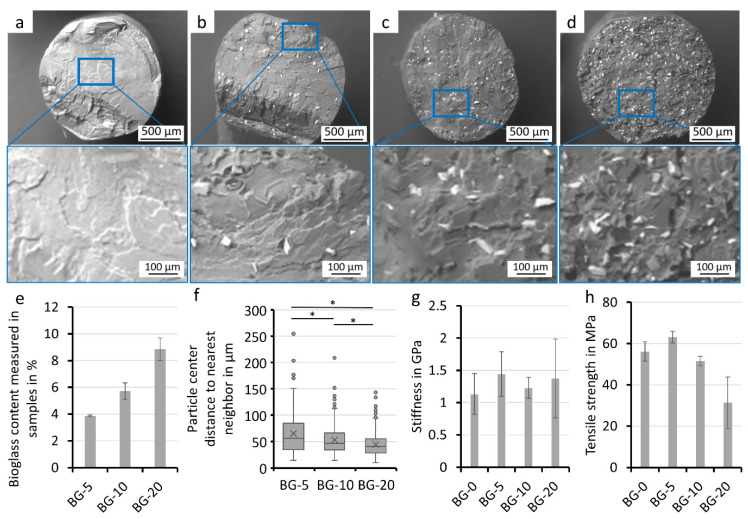
Analysis of the composite filament. SEM-Images of the custom-made PLA-bioglass composite filaments and their close-ups: BG-0 (**a**), BG-5 (**b**), BG-10 (**c**), BG-20 (**d**). The PLA matrix is visible in grey; bioglass particles in white. The area of bioglass particles in the cross section was analyzed via a threshold, and bioglass weight in the filament was calculated using the specific densities of PLA and bioglass (Means ± SD, *n* = 3) (**e**). Distance between bioglass particle centers and their nearest neighbor is shown (two samples per bioglass content with at least 231 particles were analyzed, * indicates significant differences of *p* < 0.05) (**f**). Stiffness (**g**) and tensile strength (**h**) of the custom-made filaments are presented (Tensile Testing, Means ± SD, *n* = 3).

In order to measure bioglass content and the distribution of the bioglass particles for quality monitoring, SEM images of the filament cross section were analyzed using automatic particle detection in ImageJ. The images show bioglass particles in white in the PLA matrix (Figure 2a–d). The oval shape is due to the deformation from creating the cross section. The bioglass fraction of each of the three cross sections was determined with automated image processing and calculated as the respective weight fraction (Figure 2e). As the bioglass content of the starting material increased, the percentage of bioglass particles in the cross section increased, demonstrating low variation. Interestingly, the bioglass content measured in the extruded filament deviated considerably from the values of the starting materials with bioglass contents of 0%, 5%, 10%, and 20% (*w*/*w*) (Figure 2e).

The distribution of bioglass particles was analyzed to evaluate the homogeneity of the filament (Figure 2f). The distance to the nearest particle neighbors center was determined with ImageJ. As expected, the distance between the particles decreased with increasing bioglass content. The distance of the particles the filaments (BG-5, BG-10 and BG-20) hereby differs significantly.

The mechanical properties of the bioglass filaments were determined using uniaxial tensile testing (Figure 2g,h). As the bioglass content increased, the maximal tension decreased, although no clear trend for the stiffness of the filaments was apparent. No significant differences could be detected either. Depending on particle matrix bonding, elastic modulus can be impacted. The lack of clear variation indicates sufficient bonding between the two components.

### 3.3. Processing of Composite Filament

**Figure 3:**
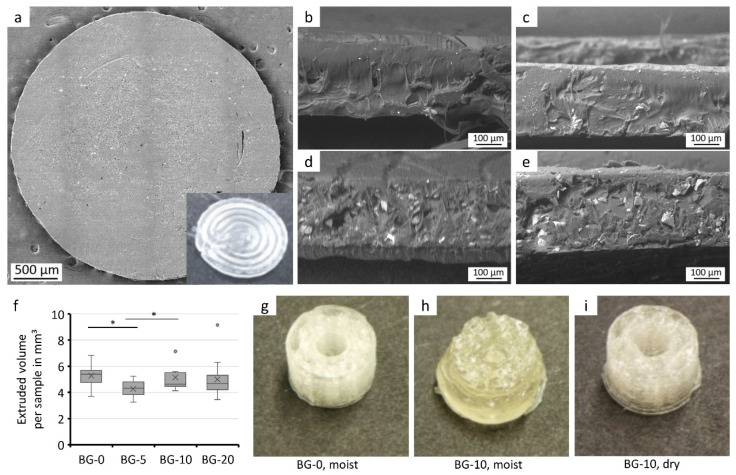
Composite filament processing via FFF. SEM and photographic image of a simple cylindrical structure printed via FFF with two layers (**a**). SEM images of the cross section of the cylindrical structure printed with BG-0 (**b**), BG-5 (**c**), BG-10 (**d**) and BG-20 (**e**). Extruded volume per cylindrical structure, calculated using the weight and the respective bioglass contents of the composite filaments (**f**) (*n* ≥ 12, * indicates significant differences of *p* < 0.05). Design printed with moist filament without (**g**) and with bioglass content (**h**) and printed with dried filament with bioglass content (**i**).

**Figure 4:**
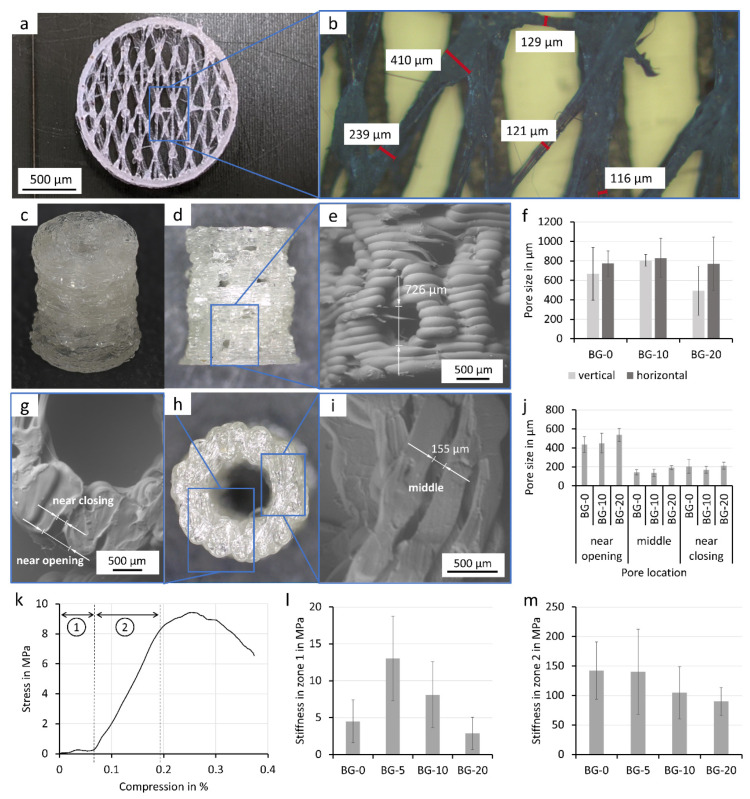
Characterization of FFF printed porous scaffolds. Scaffold design resembling spongiosa of the bone printed with BG-20 (**a**) and a microscopic image with measurements of the strand diameters (**b**). Isometric view on a pillar structure with three different porous features (printed with BG-0 filament) (**c**). Photographic (**d**) and SEM image (**e**) of the side view of the porous pillar with visible pores through the wall and an analysis of their size (Means ± SD, *n* ≥ 5) (**f**). Photographic (**h**) and SEM images (**g**,**i**) of the top view of the porous hollow pillar with visible pores in the wall and an analysis of their size depending on their location (Means ± SD, *n* ≥ 3) (**j**). Exemplary mechanical characterization in compression of the porous pillar structure with two zones with distinct mechanical properties (**k**). The stiffness of the pillar structures printed with the composite filament are analyzed for zone 1 (**l**) and for zone 2 (**m**) (Means ± SD, *n* = 5).

**Figure 5:**
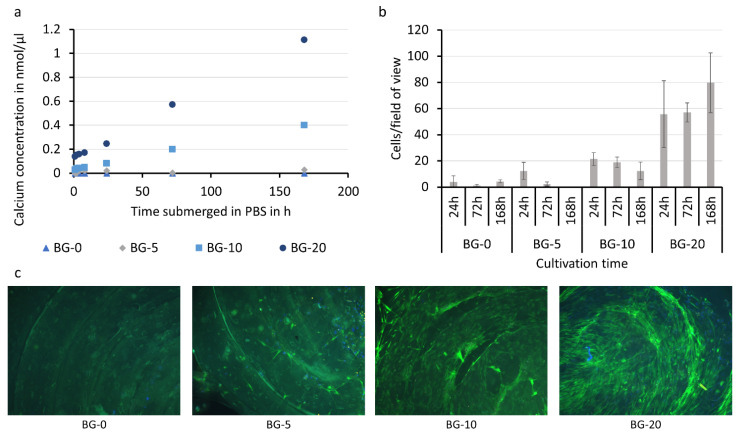
Bio-chemical properties of the PLA-bioglass composite filaments. Calcium release of PLA-bioglass composite samples submerged in PBS (**a**). Adhesion and proliferation of MSC on composite samples (Means ± SD, *n* = 3) (**b**) and exemplary fluorescence images of MSC seeded on the samples after 24 h (staining green: calcein, blue (cell nuclei): DAPI) (**c**).

Simple cylindrical structures with two layers, a 300 µm height, and a 5 mm diameter (Figure 3a) were used for the analysis of processability of the composite filament (Figure 3 and Figure 4) and bio-chemical analysis (Figure 5). Hereby a nozzle diameter of 0.4 mm was used, as a smaller diameter results in a clogged nozzle. The picture of the simple cylindrical structure clearly depicts the printing path visible on the top side, while the SEM image of the bottom side (which was in contact with the printing bed) shows a smooth surface.

In the cross sections of the printed samples (Figure 3b–e), the two layers cannot be visibly separated. As expected, the bioglass particle concentration in the cross section increased along with the bioglass content of the filament used for fabrication. The particle distribution was homogeneous. Between the bioglass particles and the PLA-matrix there was no visible gap.

The simple cylindrical structures were printed as a sample geometry for processability assessment using filaments containing different bioglass concentrations. The extruded volume of the filament was then calculated using the two densities (of PLA and bioglass) and their corresponding weight percentages in the filament (Figure 3f). The samples containing bioglass have a lower extruded mean volume. However, significant differences can only be detected between the BG-0 and BG-5 and between the BG-5 and BG-10 samples.

Processability of the composite filament not only depends on the bioglass content, but on the water content of the filament (Figure 3g–i). Moist composite filaments (Figure 3h) showed a lower shape fidelity and lower resolution in comparison with wet filaments without bioglass (Figure 3g) and dried composite filaments (Figure 3i). Samples printed with dried composite filaments (for 12 h at 40 °C) showed similar shape fidelity compared to filaments without bioglass content.

### 3.4. FFF Printed Porous Scaffolds

Complex geometries with a pore size gradient and different functional units were developed to achieve improved bone defect healing. The first (Figure 4a,b) resembles the structure of the highly porous cancellous bone with its fine trabecular structures. These fine structures were printed using a BG-20 filament and a nozzle with 0.4 mm diameter. To create these small structures, a special printing technique utilizing reduced extrusion rates and string drawing was employed. To our knowledge, we are the first to use this technique with bioglass-laden PLA-composite filaments. Strand diameters between 500 and 100 µm and features within about ½–¼ of the nozzle diameter could be produced.

A porous pillar structure was designed as part of a modular approach to repair large bone defects [28] (Figure 4c). For this purpose, a cylindrical structure (5 mm outer diameter and 2 mm inner diameter) that integrates multiple levels of porosity was generated (Figure 4c). To offer space for the invasion of vasculature, macroscopic pores down to 700 µm were integrated into the outer wall of the structure (Figure 4d–f). In addition, the novel printing technique enabled the fabrication of wall segments with intricate microporosity in order to increase cell adhesion (as demonstrated in Figure 5c). Thus, highly porous inner and outer wall segments were created with pore diameters ranging down to 150 µm (Figure 4g–j). Our study reveals that the size of macroscopic (Figure 4f) as well as microscopic pores (Figure 4j) was not affected by the supplementation of the polymer with different bioglass.

The cylindrical structures were characterized for their mechanical properties with a unilateral compression test. Interestingly, the integrated macro- and microporous features led to a differentiated mechanical behavior of the construct that could be divided into two zones of differing structure elasticity, as shown in the included plot (Figure 4k). For strain ratios ranging from 0% to 7% compression, the constructs exhibited a lower structural elasticity (5–15 MPa) that was over 10-fold greater than the highly strained samples with 7% to 19% compression (100–150 MPa). Interestingly, both zones showed opposite behavior with respect to increasing bioglass content (Figure 4l,m). In the first zone, a mixture of minor deformation and pillar sample alignment occurred, demonstrating that low bioglass content (BG-5) increases the stiffness. With increasing bioglass content (BG-10, BG-20), the stiffness decreased. In the second zone, however, larger deformations were observed in the macropores and the stiffness decreased with increasing bioglass content. Except for the first zone between BG-0 and BG-5, the mechanical properties of the specimen did not differ significantly from the BG-0 sample, respectively.

### 3.5. Bio-Chemical Characterization of PLA-Bioglass Samples

Finally, the bio-chemical properties of the bioglass filament were assessed (Figure 5). A calcium release study was performed by immersing the samples in PBS (Figure 5a). As expected, the sample without bioglass content did not release calcium. The samples with bioglass content released calcium shortly after immersion, as shown by the data after 1 h. The released amount of calcium increased along with the bioglass content of the filament over time. For instance, samples with high bioglass content released more than 1 nmol of calcium per µL in the course of 168 h, while only 5% or 10% bioglass supplementation (BG-5 and BG-10) led to a strongly reduced calcium release of 0.4 and 0.03 nmol/µL over the same time period. The samples showed a prolonged release of the bioglass over 7 days. It is expected that additional bioglass would be released as the PLA matrix degrades and particles in the middle regions of the composite become exposed.

Cell growth on the samples was examined with fluorescence microscopy (Figure 5b,c). It was shown that increasing the bioglass content strongly improved the cell adhesion potential (Figure 5c). Cell adhesion was quantified at three different time points (24 h, 72 h, and 168 h). The control samples without bioglass (BG-0) and BG-5, expressed the lowest cell count, which even decreased over time. In contrast, PLA with elevated bioglass contents (>10%) indicated a strongly improved primary cell adhesion (24 h after cell seeding). However, the best results were achieved with BG-20 samples. These not only exhibited significantly heightened primary cell adhesion, but also supported the adhesion of proliferated cells. In sum, it was shown that the cell adhesion potential of BG-20 was more than 10 times higher than BG-0 (13.9 times for 24 h, 57 times for 72 h, and 18.5 times for 168 h). Besides the effect of bioglass supplementation, the study revealed the effect of microgrooves, which were created as an artifact on the sample surface during the printing process, on cell adhesion. In all samples (with or without bioglass), an agglomeration of cells could be identified alongside the circular oriented valleys between individually printed polymer strands. With increasing BG-content, the described observation is emphasized. In future work, this effect could be used to generate scaffolds with intricate micro-signatures that enable the controlled orientation of cells along pre-defined patterns.

## 4. Discussion

In this work we demonstrated a solvent-free fabrication of PLA-bioglass composite filament for the treatment of large bone defects. We presented the processing via FFF in highly delicate structures and confirmed the calcium release and its effect on MSC adhesion and proliferation.

This study demonstrated that fused filament fabrication has several advantages over microextrusion-based approaches: the filaments are solvent-free, storable, and allow easy quality control before fabrication of the structures. Furthermore, it allows for high throughput bioproduction, which can be supplemented with a modular system so that the correct geometry for the individual defect can be assembled in the operating room and the defect can be treated quickly [28].

We were able to create composite filaments with a simple extrusion process while maintaining high bioglass contents. The quality of the composite filaments produced in this work is largely dependent on the manufacturing parameters (Figure 1). The filament ovality and diameter, which are important parameters in FFF printing, are influenced by the temperature profile in the extruder. The homogeneity of the bioglass particles in the filament, for its part, depends on the amount of bioglass-PLA pellet mixture added, as high volumes segregate in the hopper, which cannot be compensated by the homogenization of the extruding screw. Quality assessment of this study also revealed that the bioglass content of the manufactured filament differs from the bioglass concentration of the starting materials. To determine the volumetric content of the filaments as an approximation, an automated detection of the particles in the cross section of the filaments was used. Hereby some simplifications were made, which lowered the accuracy of the results. It was assumed that the particles were symmetrically shaped and the break point was randomly located in the filament, which would result in a normal distribution of the particle’s cross-sectional area. However, the particles are non-symmetrically elongated in shape and aligned in the extrusion direction. The break point is usually not random, since the particles serve as the starting point for the break. Moreover, when manufacturing small quantities (approx. 20 g) of composite filaments, the initial phase of the extrusion process (in which pure PLA is used) leads to a reduction in the bioglass content of the subsequent composite filament (Figure 2). By producing larger quantities of the filament and discarding the transition material, this effect was circumvented in following tests. However, the method for determining the resulting values of bioglass content in the composite filaments can still be used as a reference for quality monitoring new filament.

In this paper, influences of bioglass particles and methods for processing the composite filament were presented. The rheological and therefore printing behavior of PLA was influenced by moisture (Figure 3g–i) [29,30]. This effect was noticeably enhanced by the highly hygroscopic nature of the bioglass due to its high alkali content [11,31]. The impact of the undefined melting behavior can be circumvented by employing a drying step before processing the filament and its components. Furthermore, other working groups found that due to the addition of bioglass the thermal degradation of the PLA starts at lower temperatures (from about 225 °C) [32,33]. When choosing extruding and printing temperatures thermal degradation and bonding of the layers need to be considered [34].

We were able to create composite structures with a very high resolution of about ⅓ of the nozzle diameter (approx. 120 µm). For this, a nozzle of at least 0.4 mm has to be used to avoid clogging, which confirms other studies which state a nozzle/particle diameter ratio of 6.2–7.5 is needed for sufficient volume flow [35,36,37].

We were able to successfully print structures with a bioglass content of up to 20% of the starting material with a controllable pore size gradient, which has been shown in other studies to be beneficial for cell adhesion and bone tissue development [17,24]. Furthermore, the stiffness of the BG-20 composite pillar structures at a compression over 5% (90 MPa) and of BG-20 composite filaments (1.1 GPa) is similar to the stiffness of human trabecular bone (50 MPa–24 GPa), preventing stress shielding induced bone loss after implantation [8,38,39,40]. The utilized design containing a mixture of large load-bearing structures and delicate structures can be easily altered to match the remodeling of the novel bone in the body [5].

The cross-sections of the printed samples showed a homogeneous distribution of the particles after printing and a good adhesion of the layers (Figure 3), as they could not be visually distinguished from each other. Several other studies find insufficient adhesion between the bulk material and the particles, which can be attributed to the hydrophobicity of the PLA and the hydrophilicity of the bioglass [6,8,41,42]. This manifests itself as dark zones around the particles in SEM images and leads to a decrease in elastic modulus in tensile tests [8]. Gerhard and Boccaccini and their composite theory of ceramic-laden polymer scaffolds with optimal interface bonding confirmed that good bonding leads to a strengthening of the scaffolds [8,17]. The composite of this study showed similar filament stiffness for the different bioglass contents (Figure 3); therefore, taking the other studies into account, sufficient but not ideal particle matrix bonding is assumed. An interesting approach to solve this issue in future experiments was presented by Serra et al. [43]. The group introduced the addition of PEG to the matrix material as a way to increase the hydrophilicity of the PLA-matrix and enable improved particle matrix bonding and cell attachment.

Besides hydrophilicity, pure PLA is not an ideal material for cell culture due to its degradation with acidic products (Figure 5) [10,44]. The bioglass in the composite, however, neutralizes the acidic degradation products of the PLA [44]. As the composite filament degrades, new bioglass particles are released due to the homogeneous distribution of the particles in the filament. The continuous release of bioglass of the composite filament exhibits benefits over pure bioglass, where other studies have reported the problem of too fast dissolution and resorption rates, causing cytotoxic effects and negatively affecting the balance of natural bone remodeling [13,16,45].

To investigate the biological properties of the filaments, MSCs were seeded on the composite specimens. MSCs are plastic-adherent, multipotent, non-hematopoietic cells that were selected due to their potential to differentiate along the osteogenic, chondrogenic, and adipogenic cell lineage [26]. Furthermore, the application of autologous MSCs has shown significant improvements in healing outcomes of large bone defects in various animal models (mouse [46], rat [41], sheep [42]) as well as in clinical studies [47]. In this study, the positive effect and importance of bioglass supplementation on the cell adhesion and proliferation potential was confirmed [7,8,24] (Figure 5).

In our study, bioglass-supplemented PLA samples revealed a continuous release of calcium over time. The immediate release of medium calcium levels (especially observed for BG-10 and BG-20) did not only show a strongly improved primary adhesion of seeded cells, but also favored cell proliferation and prolonged cell–matrix interaction. In addition, immediate bioglass release is particularly important as the bioglass inhibits the growth of various bacterial strains [13,14], reducing the risk of infections and implant encapsulation, which are still major issues associated with implants in regenerative medicine [45]. This is of particular interest for a future translation of the results into (pre-)clinical applications. Last but not least, the 3D-printing induced formation of microgrooves on the sample surface was shown to act as a potential niche for cell aggregation. The observed effects are in line with previous work that emphasize the influence of surface roughness on cell adhesion [48]. In the future, this phenomenon could be exploited to controllably generate cell niches or dominate cell orientation in pre-defined patterns.

## 5. Conclusions

In this work, we presented the manufacturing parameters and quality assessment of a custom-made S53P4 bioglass and PLA composite filament for FFF printing of scaffolds for the treatment of large bone defects. The filaments showed a similar diameter distribution such as commercial filaments, good printability, and homogenous particle distribution. Highly porous designs with pore sizes from 180 µm to 2 mm and strand diameters down to 120 µm were achieved, which equals about ⅓ of the nozzle size. Hereby a lower water content of the filament improved the printing results. The produced scaffolds showed promising porosity for bone defect treatment including vascularization and mechanical strength. The positive prolonged biological influence of the bioglass was confirmed by the combination of a calcium release study and MSC attachment and proliferation.

## Data Availability

The data presented in this study are available on request from the corresponding author.
